# Characterization of exceptional responders with long-term PARP inhibitor therapy in recurrent ovarian cancer: an analysis of 23 patients from Charité

**DOI:** 10.1007/s00404-026-08309-2

**Published:** 2026-02-26

**Authors:** Jacek Glajzer, Jalid Sehouli, Hannah Woopen, Elena Ioana Braicu, Joanna Baum, Jacek P. Grabowski

**Affiliations:** 1 Department of Gynecology and Obstetrics, Breast Center Ostsachsen, Klinikum Oberlausitzer Bergland Zittau/Ebersbach, 02730 Ebersbach, Germany; 2 Department of Gynecology With Center for Oncological Surgery, Corporate Member of Freie Universität Berlin and Humboldt-Universität zu Berlin, Charité-Universitätsmedizin Berlin, Augustenburger Platz 1, 13353 Berlin, Germany

**Keywords:** Ovarian cancer, Exceptional response, Long- term survival, PARPi therapy, Maintenance therapy

## Abstract

**Objective:**

This analysis aimed to characterize exceptional responder with long-term PARP inhibitor therapy (ExR-LT) in platinum-sensitive recurrent ovarian cancer.

**Methods:**

This analysis included ExR-LT. ExR-LTs are defined as patients that received a continuous maintenance therapy for recurrent ovarian cancer with olaparib or niraparib for at least 5 years and showed an exceptional response. Exceptional response was defined as progression-free survival (PFS) of at least 5 years. This analysis has a retrospective and descriptive character.

**Results:**

23 patients were included. The median duration of PARPi therapy was 7.1 years (range 5.3; 10.5). The longest treatment duration was reached in the BRCA1 mutation (BRCA1m) cohort with a mean duration of 8 years (range 5.3; 10.5 years). The majority of patients (16 patients, 69.7%) reported adverse events (AE) during PARPi therapy. 12 patients (52.2%) had mild AE (CTCAE 1 or 2), 4 patients (17.4%) reported more severe AE (CTCAE 3). 14 patients needed a dose reduction due to treatment-related AE (60.1%). The most common indications for dose reduction were anemia (17.4%), headache and limb pain (17.4%), and fatigue (13%). Four patients (17.4%) required an interruption of PARPi therapy. Ten patients received a dose reduction within the first 6 months and two patients after one and 1.3 years of PARPi therapy. No dose adjustments were necessary between 1.5 and 4 years. After 4 years, 3 patients (13%) received a late dose reduction. 8.6% had another cancer diagnosed before, 4.3% simultaneously, and 13% after the ovarian cancer diagnosis.

**Conclusion:**

ExR-LTs present with heterogenic clinical and genetic characteristics. Clinical management is complex because of a high rate of AE and need of dose reductions at various points in time. Close monitoring for AE, recurrences and secondary malignancies must be carried out throughout the entire time of treatment.

## What this study add to the clinical work

**Table:** 

This study shows that patients with unfavorable clinical factors or BRCA wild type have the chance to become ExR-LT. During follow-up, ExR-LT require close monitoring—initially for adverse events, and later for adverse events with delayed onset, recurrence and secondary malignancy.	

## Introduction

Ovarian cancer is the eighth most common cancer type in women worldwide [1]. Therapy consists of a multimodal approach with a multivisceral debulking surgery followed by a platinum-based chemotherapy and a maintenance therapy. Although the therapeutic strategies are evolving, ovarian cancer has the highest mortality rate among gynecological cancers with a 5-year survival of 30–40% and 10-year survival of 17% [2].

Reaching overall survival of at least 5 years makes the patient a “long-term survivor with gynecological cancer” as defined by the Gynecologic Intergroup Consensus Guideline [3]. Beneficial factors for reaching long-term survival are inter alia a young age at first diagnosis, non-serous histology, low grading, no residual tumor after debulking surgery and response to platinum-based chemotherapy [2, 4]. In platinum-sensitive recurrent ovarian cancer, a long-term benefit on progression-free survival (PFS) can be also achieved through a maintenance therapy with poly(ADP-ribose) polymerase inhibitors (PARPi). This benefit was shown in the final analysis of the SOLO2/ENGOT-Ov21 study at a median follow-up of 65.5 months for the olaparib cohort and 64.5 months for the placebo cohort [5]. Thereby, the median time to first subsequent therapy or death (TFST) was longer with olaparib (27.4 months) than placebo (7.2 months; hazard ratio (HR) 0.37, 95% confidence interval (CI) 0.28–0.48). A similar effect was observed with niraparib in the ENGOT-OV16/NOVA study at a median follow-up of 75 months [6]. The median TFST was 19.1 months for niraparib and 8.6 months for placebo in patients with germline BRCA mutation (gBRCAm) (HR 0.57; 95% CI, 0.41–0.78) and 12.4 months vs. 7.4 months in the non- gBRCAm cohort (HR 0.58; 95% CI, 0.45–0.74). For ovarian cancer recurrence, PARPi therapy starts after response to platinum-based chemotherapy and will be continued until progression or intolerable toxicity [7, 8]. These factors lead to a growing number of patients with a PARPi intake over several years. The prospective non-interventional study C-Patrol analyzed real-world data on patients with recurrent ovarian cancer and olaparib maintenance [9]. It showed that 10% of the 267 included patients received olaparib for 5 years or longer. Although attention to long-term intake of PARPi is increasing, data are lacking.

This analysis is the first to characterize a patient cohort with exceptional response and long- term PARPi therapy. The focus lies on patients’ characteristics, clinical management of PARPi therapy including tolerability, dosage, and adverse event (AE) management and long-term aspects including secondary malignancies and recurrence. The aim is to describe the cohort and identify factors that lead to exceptional response to PARPi.

## Methods

This retrospective analysis included exceptional responder with long-term PARPi therapy (ExR-LT) in platinum-sensitive recurrent ovarian cancer treated at the department of gynecology at Charité Universitaetsmedizin Berlin between 01.01.2015 and 30.11.2025. ExR-LTs are defined as patients that received a continuous maintenance therapy for recurrent ovarian cancer with olaparib or niraparib for at least 5 years and showed an exceptional response. Exceptional response was defined as PFS of at least 5 years.

The data were extracted from the patients’ records. This study was performed in line with the principles of the Declaration of Helsinki. Approval was granted by the Clinical Ethics Committee at Charité Universitaetsmedizin Berlin (No. EA2/014/24). All patients had a histologically confirmed diagnosis of high-grade serous or endometrioid ovarian, fallopian tube or peritoneal cancer, and a histologically or radiologically confirmed relapse. All patients had received at least two lines of platinum-based chemotherapy and had a partial or complete response to the most recent platinum-based chemotherapy. Response was evaluated using the modified Response Evaluation Criteria in Solid Tumors version (RECIST) 1.1. or CA-125 levels. All patients had been receiving regular follow-up appointments during the treatment period. Safety was analyzed in all patients. AEs were classified according to the National Cancer Institute Common Terminology Criteria for Adverse Events (CTCAE) version 4.0. Classification was carried out retrospectively using the patients` records. In accordance with the journal’s guidelines, we will provide our data for independent analysis by a selected team of the editorial for the purposes of additional data analysis and the reproducibility of this study in other centers if such is requested.

Analysis was mainly descriptive. Continuous data were described using mean and range. Categorical data were described with absolute frequencies and percentages.

## Results

### Patient characteristics

23 patients were included in this analysis. The patients’ characteristics are shown in Table 1. At the time of PARPi therapy onset, 18 patients had the first (78.3%), three patients the second (13%), and two patients the third recurrence (8.7%). The tumor localizations at recurrence were as follows: peritoneal recurrence (13 patients, 56.5%), lymphogenic recurrence (3 patients, 13%), mixed peritoneal and lymphogenic recurrence (3 patients, 13%), mixed peritoneal/lymphogenic recurrence with distant metastases (3 patients, 13%). The recurrence localization of one patient was unknown (4.3%).

**Table 1:** Patient’s characteristics at first diagnosis and at recurrence before PARPi intake

Parameter	At first diagnosis (*n* = 23)	At recurrence before PARPi therapy (*n* = 23)
Age (years)	52.9 (36; 69)	57.2 (40; 72)
Diagnosis, *n* (%)		
Primary ovarian cancer	21 (91.3%)	
Fallopian tube cancer	2 (8.7%)	
FIGO stage, *n* (%)		
IIA	1 (4.3%)	
IIB	0	
IIC	1 (4.3%)	
IIIA	1 (4.3%)	
IIIB	2 (8.7%)	
IIIC	14 (60.9%)	
IVA	2 (8.7%)	
IVB	1 (4.3%)	
Unknown	1 (4.3%)	
Histological diagnosis, *n* (%)		
High-grade serous papillary	22 (95.7%)	
Endometrioid	1 (4.3%)	
Mutational status, *n* (%)		
sBRCA1	1 (4.3%)	
gBRCA1	9 (39.1%)	
gBRCA2	6 (26%)	
gBRCA1 and gBRCA2	1 (4.3%)	
MSH2	1 (4.3%)	
RAD51D	1 (4.3%)	
BRCAwt	4 (17.4%)	
Debulking surgery, *n* (%)		
Yes	23 (100%)	15 (65.2%)
No	0	8 (34.8%)
Surgery type, *n* (%)		
Debulking surgery	16 (69.6%)	15 (65.2%)
Interval surgery	2 (8.7%)	0
Completion surgery	5 (21.7%)	0
No operation	0	8 (34.8%)
Macroscopic residual tumor, *n* (%)		
No macroscopic residual tumor	21 (91.3%)	15 (65.2%)
< 2 cm	0	0
> 2 cm	2 (8.7%)	0
No operation	0	8 (34.8%)
Adjuvant therapy, *n* (%)		
Carboplatin/paclitaxel	21 (91.3%)	2 (8.7%)
Carboplatin/gemcitabine	0	4 (17.4%)
Carboplatin/pegylated doxorubicin	1 (4.3%)	17 (73.9%)
Carboplatin/docetaxel	1 (4.3%)	0
HIPEC	1 (4.3%)	0
Radiation	1 (4.3%)	0
Catumaxomab intraperitoneal	1 (4.3%)	0
Maintenance therapy, *n* (%)		
Bevacizumab	12 (52.2%)	0
None	11 (48.8%)	0
Olaparib	0	16 (69.6%)
Niraparib	0	7 (30.4%)
Chemotherapy line, *n* (%)		
2		18 (78.3%)
3		3 (13%)
4		2 (8.7%)

### Survival data

Median duration of follow-up and overall survival were 7.1 years (range: 5.3; 10.5 years). The follow-up time is equivalent to overall survival as all patients were still alive at the time of last follow-up. The median duration of maintenance therapy with PARPi equals the PFS and amounted to 6.8 years (range: 5.0; 10.5 years).

### Safety and clinical management

**Fig. 1:**
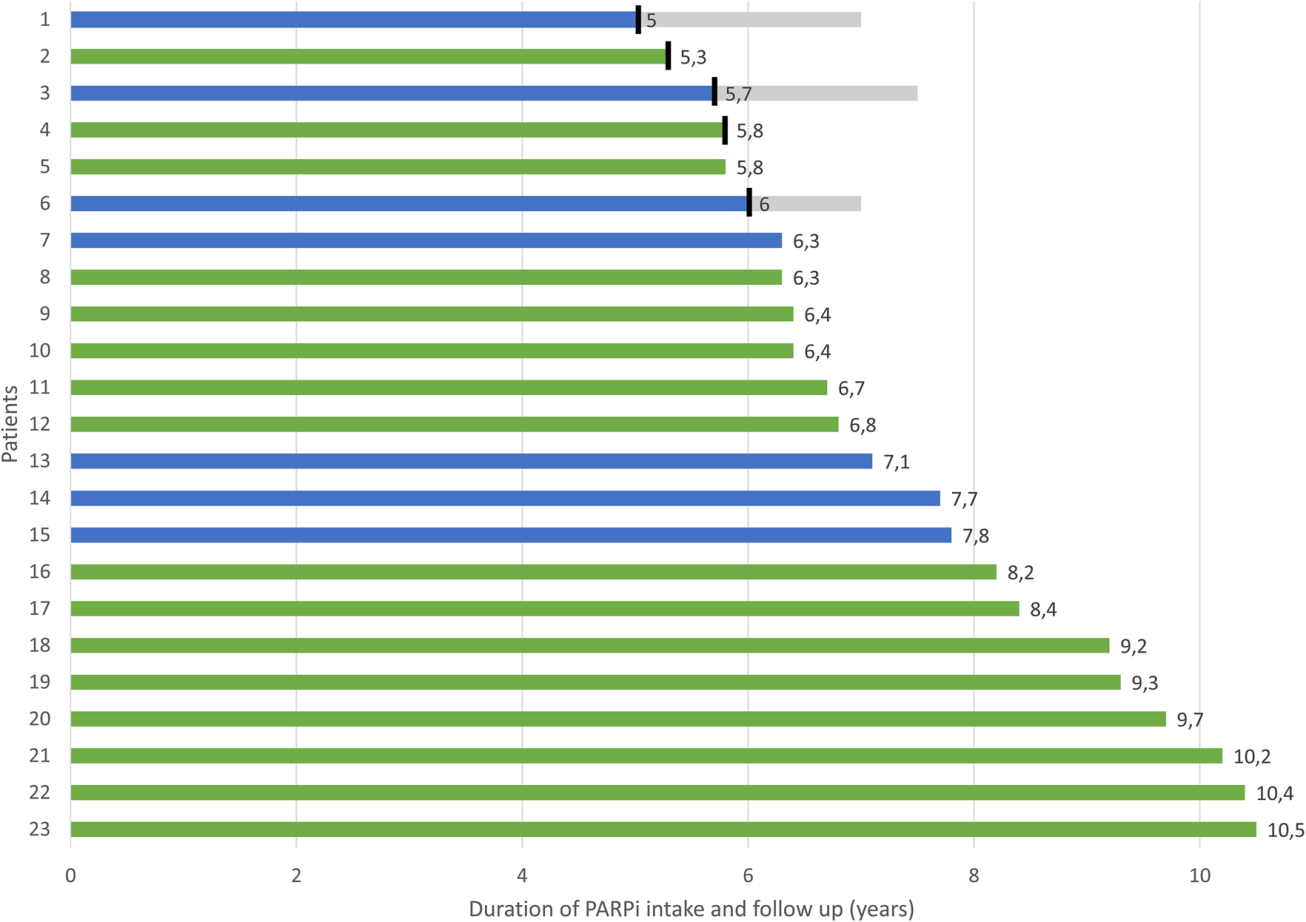
Duration of olaparib (green) or niraparib (blue) intake of each patient listed individually. Duration of follow-up after termination of PARPi therapy (gray). Termination of PARPi therapy (black strips)

16 patients (69.6%) received a maintenance therapy with olaparib and 7 (30.4%) with niraparib according to standard of care (see Fig. 1). Nine patients started olaparib as capsule formulation and seven patients as a tablet. Over the course of time, all patients had switched to tablets. Four patients (17.4%) started with a dose of 200 mg niraparib, adjusted to a body weight of less than 70 kg. One patient (4.3%) received a reduced starting dose of 200 mg niraparib due to mild leukopenia and thrombocytopenia. This patient developed high blood pressure after one month so that niraparib was further reduced to 100 mg daily. One patient (4.3%) started with olaparib 300 mg daily due to short bowel syndrome. Olaparib was increased only five weeks later to full dosage.

**Fig. 2:**
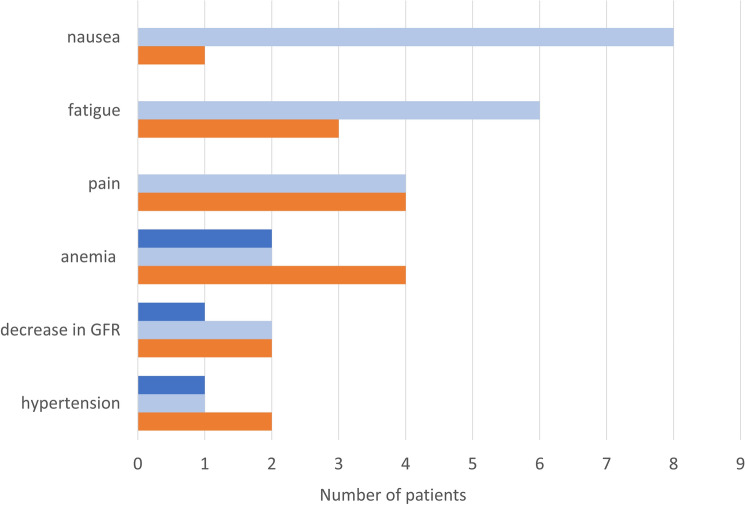
Number of patients with AE CTCAE 1 and 2 (light blue), CTCAE 3 (dark blue), indications for dose reductions (red)

The majority of patients (16 patients, 69.6%) reported AE during PARPi therapy. 12 patients (52.2%) had mild AE (CTCAE 1 or 2), 4 patients (17.4%) reported more severe AE (CTCAE 3) (Fig. 2). Nausea constituted one of the most common AE (8 patients, 34.8%). Fatigue was diagnosed in 6 patients (26%). The most frequent hematological side effect was anemia which occurred in 4 patients (17.4%). More severe AE included anemia in two patients (8.7%), a drop in the glomerular filtration rate (GFR) and hypertension in one patient (4.3%), respectively. Seven patients (30.4%) reported no side effects at all.

**Fig. 3:**
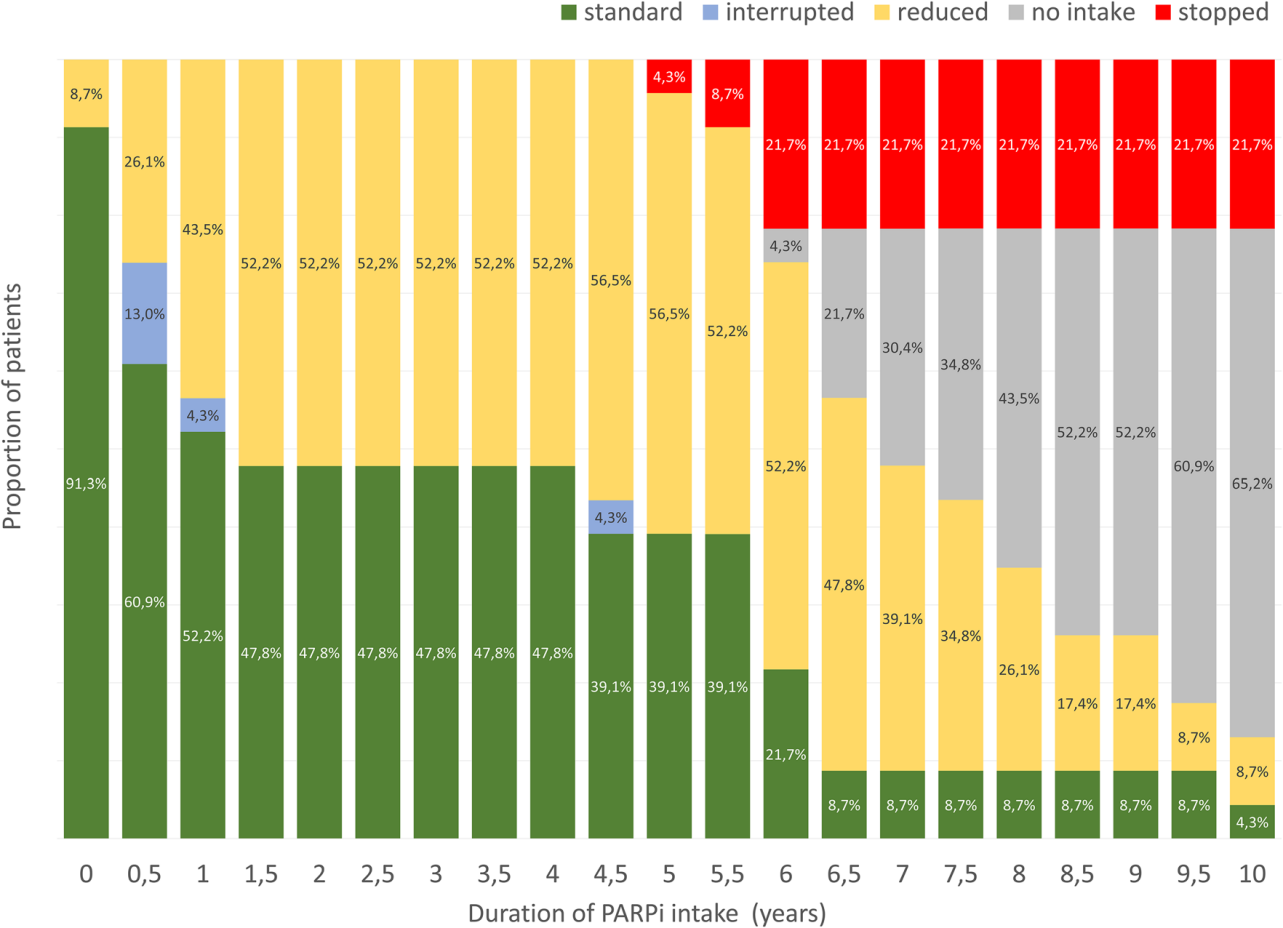
Clinical management of PARPi therapy during 10 years of follow-up: full dosage (green); reduced dosage (yellow); dosage interruption (blue); termination of PARPi intake (red); duration of intake not reached yet (gray)

14 patients needed a dose reduction due to treatment-related AE (60.1%). Three patients (13%) needed multiple dose reductions. The most common indications for dose reduction were anemia (4 patients, 17.4%), headache and limb pain (4 patients, 17.4%), followed by fatigue (3 patients, 13%), high blood pressure (2 patients, 8.7%), and decrease of GFR (2 patients, 8.7%). Ten patients received a dose reduction within the first 6 months and one patient after one and 1.3 years of PARPi therapy, respectively (Fig. 3). Three patients (13%) needed a dose interruption in this early period. No dose adjustments were necessary between 1.5 and 4 years. After 4 years, two patients (8.7%) required their first dose reduction and one patient (4.3%) a second dose reduction. Dose reductions were permanent in 92.1%.

Four patients (17.4%) required an interruption of PARPi therapy. The interruptions were performed between two weeks and one year after therapy onset. Reasons for single interruptions were an infection with cytomegalovirus, fatigue, and hypertension. Only one patient needed several dose interruptions throughout the therapy due to repeatedly decreasing GFR. All treatment interruptions resulted in a reduction in dosage. PARPi therapy had to be discontinued in 5 patients (21.7%) due to recurrence. Those patients had a total treatment duration of 5, 5.7, and 6 years with niraparib and 5.3 and 5.8 years with olaparib.

### Other malignancies

Before starting PARPi therapy, 3 (13%) patients had been diagnosed with another cancer. Thereof, two patients (8.7%) had a breast cancer diagnosis 5.6 and 11.2 years before and one patient (4.3%) 4.6 years after the diagnosis of ovarian cancer. One patient (4.3%) was diagnosed with lobular carcinoma in situ (LCIS) one year before the diagnosis of ovarian cancer. One patient (4.3%) had simultaneous endometrial cancer. During PARPi therapy, one patient (4.3%) developed urothelial carcinoma and one patient (4.3%) squamous cell carcinoma of the jaw, 4.9 and 7.6 years after PARPi onset.

### Mutational status

All patients except for one received a somatic or genetic testing. The majority of patients had a somatic or germline BRCA1m cohort (10 patients, 43.5%), followed by germline BRCA2m (6 patients, 26.1%) and no mutation (BRCAwt) (4 patients, 17.4%) (see Table 1). One patient (4.3%) had a mutation in the RAD51D, one (4.3%) in the MSH2 gene and one patient (4.3%) had a simultaneous gBRCA1m and gBRCA2m. The longest treatment duration was reached in the BRCA1m with a mean duration of 8 years (range: 5.3; 10.5 years). All three patients that reached a therapy duration of more than 10 years, had a BRCA1m and maintenance with olaparib. Mean treatment duration was 7.5 years (range: 6.3; 9.7 years) in the BRCA2m cohort and 6.1 years (range: 5; 7.7 years) in the BRCAwt cohort. The patients with MSH and RAD51D mutation had a treatment duration of 7.8 and 6.4 years. Three patients without mutation and two patients with BRCA1m developed a recurrence and terminated PARPi therapy.

Regarding the secondary malignancies, all patients with breast cancer and LCIS had a BRCA1m. The squamous cell carcinoma of the jaw appeared in the BRCA2m cohort. One patient with MSH2 mutation had a simultaneous endometrial carcinoma and developed a urothelial carcinoma during PARPi therapy.

Eight patients (34.8%) had a PFS and PARPi therapy duration of 8 years or more. The longest duration of PARPi therapy was 10.5 years. Seven of those patients had no residual tumor after primary surgery. One patient had a residual tumor of more than two centimeters. Four patients had the first and two patients the second and third recurrences, respectively. Seven patients had a complete remission after last chemotherapy, one patient had a radiological stable disease. All eight patients started on a full dosage of olaparib as capsule formulation. Five out of eight patients needed a dose reduction. Three patients needed a dose reduction within the first 3 months, one patient after 1 year and 4.6 years, respectively. Five patients had a BRCA1m, and three patients had a BRCA2 mutation.

## Discussion

### Summary of main results

This analysis is the first to characterize ExR-LT. The majority of the patients had PARPi-related AE and needed a permanent dose reduction. The timing of dose reduction varied and can be divided in three time periods. Most patients received a dose reduction within the first 6 months, no dose adjustment was necessary between 1.5 and 4 years, after 4 years 3 patients required a dose reduction. Patients with BRCA1m reached the longest PFS. Although the majority of patients had a BRCA mutation, one-fourth of the patients harbored another or no mutation at all.

### Results in the context of published literature

The literature was constantly evolving the concept of special benefit under PARPi therapy by prolonging the PFS in the definition. Initially, Swisher et al. defined “long-term response” to rucaparib as a partial or complete response of more than one year [10]. O’Malley used the term of “exceptional benefit” to rucaparib therapy defined as PFS of 2 years or more [11]. Characteristics of patients with “exceptional benefit” included no measurable tumor at the start of rucaparib, a long platinum-free interval after the previous platinum-based chemotherapy and certain genetic mutations, such as BRCA, RAD51C, RAD51D, and loss of heterozygosity (LOH) [10, 11]. Graybill et al. found similar criteria for “long-term progression-free survival” defined as a PFS of 2 years or longer to niraparib maintenance therapy in the first line [12]. The criteria contained BRCA1m/BRCA2m/HRD status, FIGO stage at primary diagnosis and number of baseline non-target lesions [12]. Recently, Haggstrom et al. conducted a survey on the recommended duration of PARPi therapy in recurrent ovarian cancer using the term of “exceptional responders” [13]. This term included patients with a PFS of 5 years or longer irrespective of duration of PARPi intake [13]. Another study that analyzed real-world data of long-term PARPi therapy was C-Patrol [9]. Marmé et al. observed that longest overall survival was achieved by patients with no residual tumor after the current relapse surgery, complete response to current platinum-based chemotherapy and BRCA2m [9].

The present analysis contains a cohort with a long-term intake of PARPi and long-term PFS of at least 5 years. The criteria for beneficial response to PARPi therapy identified by the current literature have been met in most of the presented patients: the majority had an excellent upfront situation with either a serologic (14 patients, 60.9%) or radiologic complete response (7 patients, 30.4%) prior to PARPi therapy onset. Furthermore, most patients (78.2%) had one of the above mentioned favorable genetic alterations. However, it has to be pointed out that two patients had measurable tumor at the time of PARPi therapy onset with radiologic stable disease (4.3%) or radiological partial response (4.3%) and four patients (17.4%) did not harbor any favorable genetic mutation. Human recombination deficiency (HRD) was not examined in this cohort. O’Malley et al. comes to the conclusion that exceptional benefit is not exclusive to patients with favorable characteristics [11]. This is in line with the presented data which suggest that there are more factors than the mentioned clinical and genetic favorable characteristics that lead to exceptional response to PARPi. Another factor that might have an influence on the duration of PARPi therapy is the clinical management. In the presented cohort, 69.7% of the patients reported AE, 60.1% needed a dose reduction, and 17.4% had one or more dose interruptions. Thereby, it has to be pointed out that almost all of the patients with dose reductions stayed on the lower dose during the further course of treatment and were still able to reach exceptional response. In 3 patients, dose adjustments were necessary after 4 years of PARPi intake. Moreover, five patients developed a recurrence after 5 years of PARPi therapy. From this it can be concluded that ExR-LT need a long-term follow-up with thorough examinations .

The clinical management also needs to be adapted to the risk of other malignancies. Woopen et al. observed in a study of 225 long-term survivors with ovarian cancer (LTS), defined as OS of more than 8 years after ovarian cancer diagnosis, that 16% of the LTS had been diagnosed with more than one cancer either before, simultaneous or after the ovarian cancer diagnosis [14]. A secondary malignancy was observed in 7.6% of the LTS. These numbers correspond to the presented cohort, where 8.6% had a cancer diagnosed before, 4.3% simultaneously, and 13% after the ovarian cancer diagnosis. The high proportion of patients with further malignancies before the start of PARPi therapy highlights the increased risk associated with the mutational status. Kuchenbaecker et al. showed that BRCA1m carriers have a cumulative breast cancer risk to the age of 80 years of 72% and BRCA2m carriers of 69% [15]. Therefore, it is essential to offer follow-up care for the detection of cancer recurrence and late-onset AE. At the same time, cancer screenings should be routinely offered to ExR.

### Strengths and weaknesses

This analysis is limited by its retrospective nature and unicentric design. The small number of patients and the heterogeneity of the mutational status mean that no general conclusions can be drawn. Rather, this analysis aims to discover initial tendencies and correlations for future studies. Furthermore, this analysis is descriptive with lack of a comparison group, i.e. short-term responders to PARPi with rapid progression. This could lead to a distorted perception that PARPi therapy has only long-term benefits for all patients. It has to be pointed out that only a small proportion of patients with PARPi therapy reaches a PFS of more than 5 years. An isolated view on this special cohort allows a very detailed characterization of each single patient involving aspects, such as clinical management and clinical factors. It forms the basis for future studies.

### Implication for practice and future research

This analysis indicates that routine follow-ups with thorough examinations, general cancer screening, and evaluation of AE should be offered to ExR-LT and performed on a regular basis. Dose reductions must be considered even after several years of therapy. Although there are factors beneficial for reaching exceptional response, they are not exclusive. Patients with detrimental clinical factors may also reach exceptional response. Further studies on predicting factors of exceptional response and safety aspects of long-term PARPi therapy are warranted.

## Conclusion

ExR-LTs present with heterogenic clinical and genetic characteristics. Although patients with BRCA1m had the longest PFS, patients without BRCAm can still reach exceptional response. Clinical management is complex due to a high percentage of AE. Dose reductions were frequent and mostly permanent making exceptional response still possible. Long- term follow- up is necessary for detection of recurrences, late-onset AE, and secondary malignancies.

## Data Availability

No datasets were generated or analysed during the current study.
